# Assessment of multiple-opinion referrals and consults at the BC Children’s Hospital Allergy Clinic

**DOI:** 10.1186/s13223-023-00806-2

**Published:** 2023-06-14

**Authors:** Adam P. Sage, Elliot James, Megan Burke, Edmond S. Chan, Tiffany Wong

**Affiliations:** 1 Faculty of Medicine, University of British Columbia, Vancouver, BC Canada; 2 Division of Allergy, Department of Pediatrics, BC Children’s Hospital, Room 1C31B, 4480 Oak Street, Vancouver, BC V5Z 4H4 Canada

**Keywords:** Pediatric allergy, Wait-times, Multiple-opinion consults, Quality improvement

## Abstract

**Background:**

Allergic disease is on the rise. Waitlists for specialists are long, and many referred patients have already received prior allergic assessment, either by a certified Allergist, Primary Care Provider, or other Specialist. It is important to understand the prevalence and motivating factors for multiple-opinion referrals, to deliver timely assessment for patients with allergic disease.

**Methods:**

A retrospective chart review of demographic information, number of previous consultations, and motivation for new consults and multiple-opinion referrals, of pediatric patients aged 8 months–17 years to BC Children’s Hospital Allergy Clinic from September 1, 2016–August 31, 2017, was performed. Referral data including reason for referral or multiple-opinion, primary allergic concerns, and others, from referral forms and consult notes were accessed through local Electronic Medical Records and subsequently analyzed for trends in categorical variables to assess the rationale for and impact of multiple-opinion referrals to our clinic.

**Results:**

Of 1029 new referrals received, 210 (20.4%) were multiple-opinion referrals. Food allergy was the predominant allergic concern prompting further opinion (75.7%). The main rationale for seeking further opinions was wanting an assessment by a certified allergist in cases where prior consultation was performed by non-allergist specialist, primary care provider, or alternative health care provider. Of second-opinion referrals generated, 70 (33.3%) initial consultations were performed by an Allergist, whereas 140 (66.7%) were performed by a non-allergist.

**Conclusions:**

Many new consults at the BCCH Allergy Clinic are multiple-opinion assessments, contributing to long waitlists. Advocacy at the systems level through standardized referral guidelines, centralized triaging systems, and stronger support for Primary Care Providers is needed to provide better access in Canada for children needing a specialized Allergist.

*Trial registration* UBC/BCCH Research Ethics Board

## Background

In Canada, wait times are often at the forefront of the conversation of healthcare reform, especially after the COVID-19 pandemic laid bare gaps in cornerstone aspects of care, such as the lengthy wait times for surgeries and procedures, including joint replacements or diagnostic imaging [1]. Pediatric populations are particularly susceptible to the barriers imposed by an overwhelmed medical system. To address this, a national guideline for access targets has been designed for pediatric patients undergoing surgery (Pediatric-Canadian Access Targets for Surgery, PCATS), which prioritizes patients by severity of illness and assigns an associated target wait-time [2]. Provinces are struggling to meet these targets, with a recent analysis in Quebec highlighting rurality of patients, age of patients, number of visits, and referring-physician specialty as significant barriers to specialist referral in target time [3]. While current studies and national guidelines are limited, these barriers to accessing care for the pediatric population can be extended to other services outside of surgery.

Pediatric allergy serves as an important lens for examining barriers to accessing specialty care services and patient wait times, in light of the steadily rising prevalence of atopic disease in Canada, with 27.3% of Canadians aged twelve or older reporting having allergic disease confirmed by allergy testing in 2017 [4]. Canada and other developed countries have a higher burden of allergic disease relative to the developing world, due in part to genetics, unique aeroallergens, climate, and other factors such as hygiene and microbiome influences [5, 6]. However, the number of practicing pediatric Allergists in Canada has not yet increased to meet the demands of the growing population of children living with allergic disease. In 2019 there were 0.6 certified Allergists (219 total) per 100,000 people across Canada, leading to increased wait times, decreased access to care, and frustration amongst Canadian families [7].

In British Columbia, the BC Children’s Hospital (BCCH) Allergy Clinic receives over 1000 referrals per year. The current non-urgent wait list has some patients projected to wait 2 years to see an Allergist at BCCH, which is similar for community Allergists, such as Allergy Victoria, where non-urgent Pediatric referrals wait 24–28 months on average [8]. These long wait lists have direct consequences on pediatric populations: with infants at increased risk of developing food allergy due to food avoidance or conversely unnecessarily avoiding foods, or patients seeking non-evidence-based opinions [9]. Patients and their families may wait years to receive a formal diagnosis and appropriate treatment, with many seeking alternate referrals to other Allergists with shorter waitlists or other healthcare providers altogether. Multiple-opinion referrals present a challenge by further complicating specialist clinic visits, lead to diagnostic and treatment differences, and ultimately contribute to long waitlists, all of which can result in delayed treatment of patients with potentially life-threatening allergic disease.

The impact of multiple-opinion referrals is under-reported in pediatric literature, with most recent publications focusing on the oncology setting [10]. A previous systematic review of the literature examining the factors motivating patient-initiated second-opinion consults revealed common rationale to be diagnosis or treatment confirmation, dissatisfaction with previous consultation, and desire for more information [11]. While patients are noted to see value in the second-opinion, a major change in treatment plan, diagnosis, or prognosis was observed in between 10 and 62% of these visits [11]. Recent guidelines from Otolaryngology suggest referral to a specialist only under unique circumstances, as in the case of allergic rhinitis, where referrals are restricted to patients who require immunotherapy, or have inadequate response to initial therapies [12]. No studies have examined the impact of multiple-opinion referrals in allergy.

To better characterize these issues and referral patterns, we conducted a retrospective chart review for patients being referred to the BCCH Allergy Clinic, the largest pediatric tertiary centre in British Columbia. Through this, we aimed to better understand the prevalence of these referrals and motivation for these visits, to leverage these data to both reduce wait-times and improve patient satisfaction in pediatric allergy across Canada.

## Methods

A retrospective chart review was used to conduct this single-centre quality improvement study examining the frequency and context surrounding multiple-opinion referrals to the British Columbia Children’s Hospital (BCCH) Allergy Clinic. Ethics approval from the UBC/BCCH Research Ethics Board was obtained (H18-02528). All Allergist consultation letters from new referrals sent to the BCCH Allergy Clinic from September 1, 2016–August 31, 2017 were accessed through Electronic Medical Records (EMR) associated with the Allergy Clinic. This timeframe captures both new and multiple-opinion referrals prior to the large number of second opinion referrals received reflecting the demand for the initiation of oral immunotherapy in late 2017 [13].

Non-identifiable patient demographic information was retrieved by reviewing the BCCH Allergy Clinic consult notes in patient EMRs from all referrals over this timeframe and subsequently stored in a secured Excel database. Multiple-opinion referrals were defined as patients who had been seen by at least one other Allergist or other Specialist prior to being seen at the BCCH Allergy Clinic, as described in both the patient chart and referral forms. Analysis of categorical variables was conducted to characterize frequency and reasons behind multiple-opinion consults at the BCCH Allergy Clinic. Subjective data were also retrieved from consult notes in the “Reason for Consult” section as well as “Reason for Referral” of the referral forms to provide further clarification into the circumstances initiating the desire for multiple-opinion consults. Statistical significance between categorical variables were conducted using two-sample t-tests in RStudio Version 2022.02.1 + 461. Analysis of approximate billing fees per appointment was performed using the publicly-available Payment Schedule from the British Columbia Ministry of Health [14].

## Results

Table 1 summarizes the demographic and referral pattern data from the 1029 new consults received at the BCCH Allergy Clinic over the 1-year study period. Of these, 819 were first-opinion consults, leaving 210 multiple-opinion consults, representing 20.4% of total new consults. Most multiple-opinion consults had been seen for one previous allergy assessment (second opinion, n = 175, 17.0%), with 30 third opinion consults (2.91%), and five fourth opinion, consults (0.486%). Of the 210 multiple-opinion consults, 122 (58.1%) were male, and 88 (41.9%) were female, with a median age of 6 years, ranging from as young as 8 months to as old as 17 years.

**Table 1:** Summary of sample characteristics of multiple-opinion referrals to BC Children’s Hospital Allergy Clinic from September 1, 2016–August 31, 2017

	First-opinion consult	Second-opinion consult	Third-opinion consult	Fourth-opinion consult
Demographics				
Number of consults	819 (79.6%)	175 (17.0%)	30 (2.91%)	5 (0.486%)
Median age (years)	N/A	6	7.5	11
Mean age (years)	N/A	6.47	7.83	9.00
Sex				
Male	N/A	101	19	2
Female	N/A	74	11	3
Reason for referral^a^				
Mean number of atopic conditions	N/A	1.94	2.16	2.40
Eczema	N/A	70	12	3
Asthma	N/A	39	10	1
Allergic rhinitis^b^	N/A	64	14	3
Food allergy	N/A	98	20	2
Other^c^	N/A	68	9	3
Healthcare provider providing previous opinion^d^				
Pediatrician	N/A	36	10	6
Allergist	N/A	60	24	5
ENT	N/A	4	1	1
Dermatology	N/A	2	1	1
Naturopath	N/A	28	7	2

^a^According to initial referral form. It was possible for a referral to ask for multiple conditions to be assessed in one consult

^b^Includes: allergic rhinitis with and without conjunctivitis

^c^Includes: drug-related allergies; vaccine allergies; venom allergies; perioperative reactions; non-atopic dermatitis; urticaria; angioedema; anaphylaxis with unknown trigger

^d^Where provider was specified. For third and fourth-opinion consults, it is possible that separate providers from the same specialties may be captured within the same patient (e.g., three separate allergists for fourth consult patients)

Most previous consultations were observed to have been conducted by other Allergists (Table 1), followed by Pediatricians (who are consultants in BC), and Naturopaths. In some cases, opinions were sought by other specialty services, including Dermatology, and Otolaryngology. The proportion of previous consultations performed by non-Allergists was higher (140; 66.7%) when compared to Allergists (70; 33.3%). The most common type of allergy testing previously performed was skin prick testing (Table 2), accounting for 139 (53.5%) of all multiple-opinion consults received, followed by serum specific IgE testing (sIgE, n = 39, 15.0%). A small proportion of patients had no documented previous allergy testing (n = 11, 4.20%), while some had extensive testing including Vega testing (n = 6, 2.30%) or Serum IgG (n = 14, 5.40%).

**Table 2:** Type of allergy testing previously performed

Primary allergic concern	Prevalence
Skin prick	139 (53.5%)
Serum-specific IgE	39 (15.0%)
Serum-specific IgG	14 (5.40%)
Skin prick and serum-specific IgE	9 (3.50%)
Vega testing	6 (2.30%)
Applied kinesiology	2 (0.80%)
Serum IgG and Vega testing	1 (0.40%)
None	11 (4.20%)
Unknown	39 (15.0%)

**Fig. 1:**
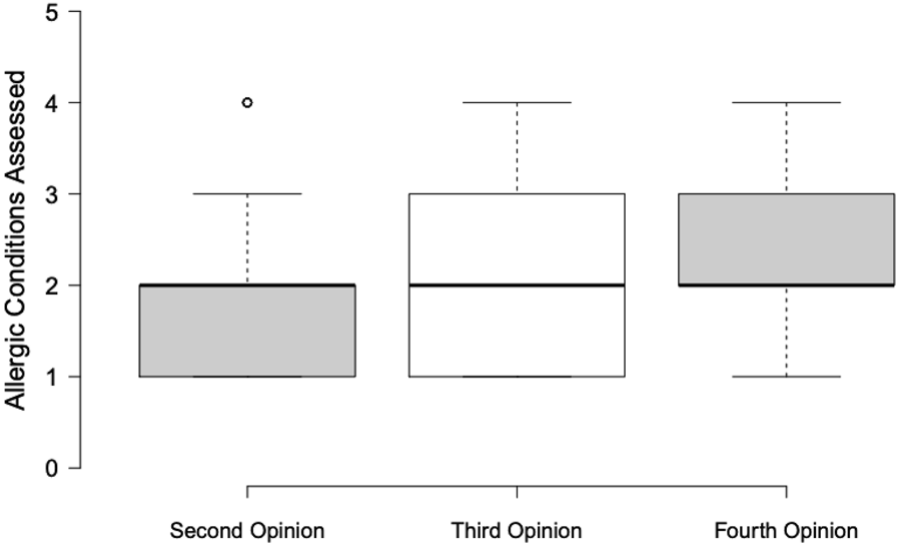
Number of allergic conditions assessed versus number of previous opinions sought. Center lines show the medians; box limits indicate the 25th and 75th percentiles as determined by R software; whiskers extend 1.5 times the interquartile range from the 25th and 75th percentiles, outliers are represented by dots

There was a trend of the average number of allergic conditions to be assessed increasing with the number of previous opinions sought by the patient and family, with an average of 1.94, 2.17, and 2.40 for second, third, and fourth opinions respectively (Fig. 1). Analysis of variance between these group means was not statistically significant (p = 0.31).

**Fig. 2:**
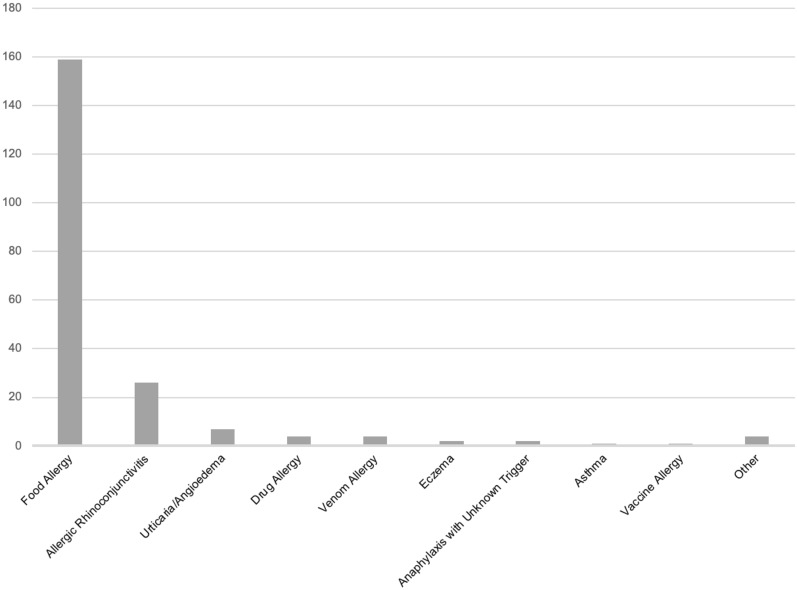
Primary allergic concern prompting another opinion. Data are represented by number of appointments indicating the associated allergy as the primary concern (y axis) as indicated on the patient’s referral form

Further, when assessing the reason for referral specifying the primary allergic concern that prompted another opinion, food allergy was significantly more prevalent than any other allergic condition (Table 3, Fig. 2, p < 0.001). These findings were mirrored when considering all atopic conditions that were assessed in the visit when considering some patients had multiple concerns to be addressed in the consult (Table 1). Other common reasons for multiple-opinions included environmental allergies and urticaria/angioedema. Asthma, eczema, and vaccine allergies were less-common reasons for multiple-opinion referrals.

**Table 3:** Primary allergic concern prompting another opinion

Primary allergic concern	Prevalence
Food allergy	159 (75.7%)
Allergic rhinoconjunctivitis	26 (12.4%)
Urticaria/angioedema	7 (3.33%)
Drug allergy	4 (1.90%)
Venom allergy	4 (1.90%)
Eczema	2 (0.95%)
Anaphylaxis with unknown trigger	2 (0.95%)
Asthma	1 (0.48%)
Vaccine allergy	1 (0.48%)
Other (perioperative reaction, pruritis)	4 (1.90%)

To assess motivations for seeking a further opinion, the primary reason for referral and rationale for second opinion aspects of referrals were assessed (Table 4). The most common reasons for seeking further opinion were patients and families specifically seeking the opinion of a certified fellowship-trained Allergist (n = 69, 32.9%), as well as dissatisfaction with previous opinions (n = 34, 16.2%). A smaller proportion of Allergist opinions were sought due to the requirement of specialized services such as venom testing that cannot be done in other offices (n = 8, 3.81%), or diagnostic uncertainty (n = 6, 2.86%). Further, some patients and families were referred to two or more providers simultaneously (n = 9, 4.29%), while some were looking for reassurance (n = 4, 1.90%), and others were referred, but sought care elsewhere in the interim as wait lists were too long (n = 6, 2.86%).

**Table 4:** Primary motivating factor for seeking another opinion

Primary motivating factor	Prevalence
Seeking opinion of certified allergist	69 (32.9%)
Dissatisfaction with previous opinion	34 (16.2%)
Preference for BCCH allergy clinic	10 (4.76%)
Referred to two providers simultaneously	9 (4.29%)
Requiring specialized services	8 (3.81%)
Diagnostic uncertainty	6 (2.86%)
BCCH allergy clinic wait list too long (sought further opinions while waiting)	6 (2.86%)
Provide reassurance	4 (1.90%)
Seeking oral immunotherapy	3 (1.43%)
Looking for more treatment options	3 (1.43%)
Previous allergist inaccessible	2 (0.952%)
Not stated	56 (26.7%)

## Discussion

This retrospective chart review study is unique in being the first to characterize the prevalence, referral patterns, and motivations behind multiple-opinion referrals to an allergy clinic. While only a single-centre study, these data serve as a representative example of the impact of multiple-opinion referrals on access to care in Canadian pediatric allergy populations. Future studies examining multiple institutions could be used to generate widespread change and improve the quality of care our patients receive.

Here, we illustrate that over twenty percent of our new consults have undergone prior allergic assessment and received advice on allergy management on at least one occasion, with most having undergone some degree of previous allergy testing (Table 2). The most common previous opinion was observed to have been provided by an Allergist working outside of BCCH Allergy Clinic, while others were seen by different specialists, revealing a possible lack of clarity in guidelines for appropriate referral patterns and a lack of support for Primary Care Providers. Two-thirds (140, 66.7%) of second-opinion consults were generated by non-Allergists, reflecting the common reason for these multiple-opinion consults to “seek the opinion of a Certified Allergist” (Table 4). The result of these multiple-opinion consults is layers of practitioner opinions and data to be reviewed with patients, more investigations, and more procedures such as oral food challenge to make an appropriate and accurate assessment and patient-centred care plan. As many patients receiving a multiple-opinion consult have already received a previous Allergic consultation, this results in a cycle of increasing wait times, prompting individuals to seek additional assessments, thereby contributing to more multiple-opinion consults.

**Fig. 3:**
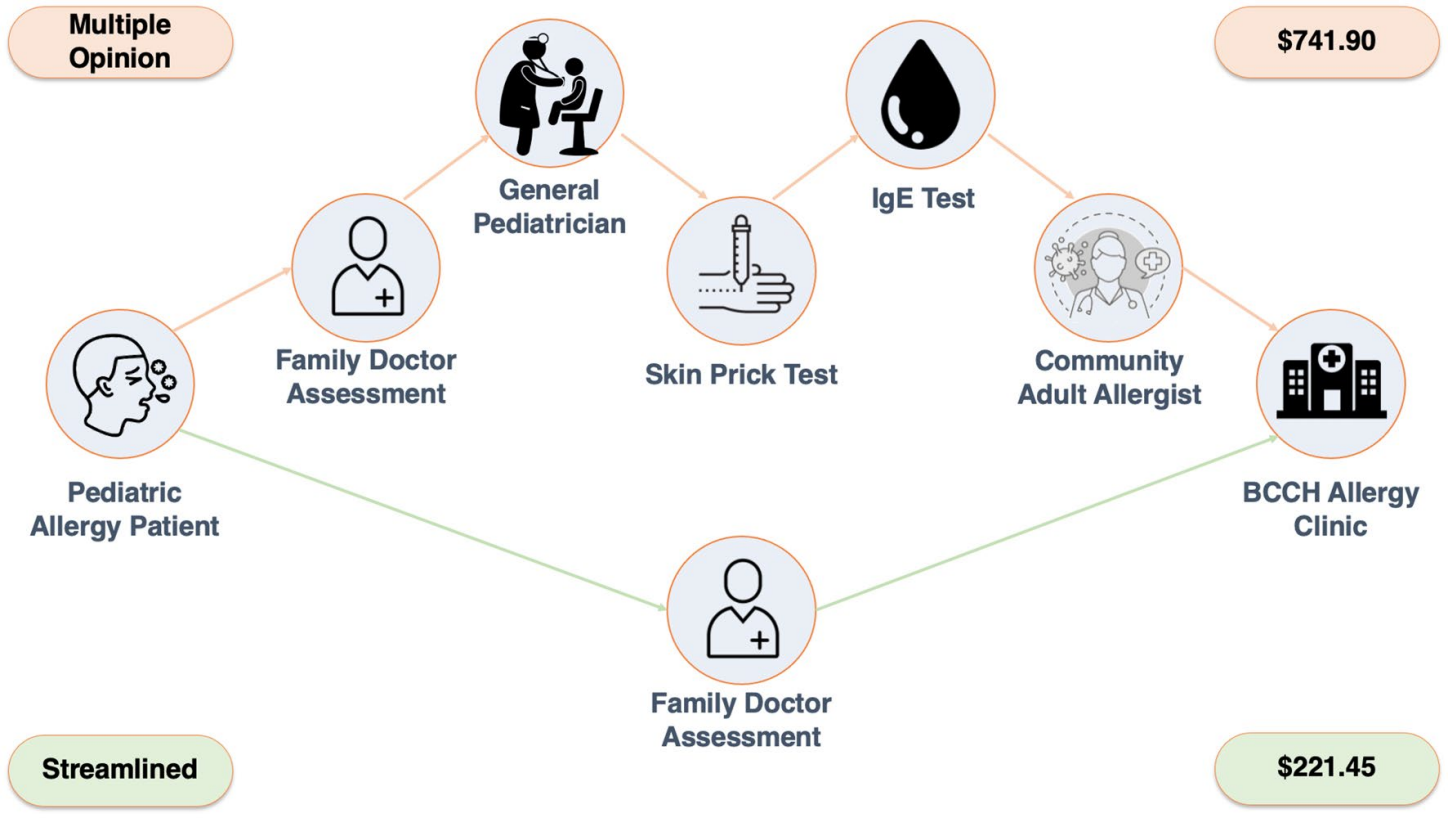
Hypothetical billing fees of multiple-opinion referrals to BCCH Allergy Clinic relative to single opinion. The “multiple-opinion” pathway wherein patients undergo multiple assessments prior to the BCCH allergy clinic consult is depicted as the upper pathway, with a hypothetical streamlined process depicted as the bottom pathway

Multiple-opinion consults pose a cost to the healthcare system, through accruing billing fees with subsequent visits to alternative providers and the possible use of costly, non-evidence based testing such as serum IgG testing [15]. For example, according to the 2021 Medical Services Payment Schedule [14], a hypothetical 6-year-old patient seen by a Family Physician (code 00100, $31.62), who is referred to a Pediatrician for consultation (code 00510, $233.15), where they receive a skin prick test for 10 allergens (code S00764, $20.15) and sIgE blood testing for 5 antigens (Laboratory code 91075, $80.65) [16], and is sent to a community adult Allergist (code 30010, $186.50) prior to BCCH allergy clinic (code 30011, $189.83) would cost approximately $741.90 in billing fees, not including costs to the family associated with time off work or travel and accommodation for out-of-town families. Further, many patients are unnecessarily avoiding foods due to positive skin or sIgE testing and will require further extended visits for oral food challenges to determine whether they are truly allergic (e.g. Oral Food Challenges, requiring further nursing support). In a streamlined process where the Primary Care Provider refers directly to a Pediatric Allergist, without the multiple-opinion consults, approximately $520.45 in billing fees alone could be saved (Fig. 3).

We observed patients sought multiple-opinions for food allergy concerns (75.7%) significantly more than any other allergic condition combined (159, 75.7% vs 51, 24.3%, p < 0.001). One possible explanation for this is the trend of a rise in awareness of food allergies among the general population. For instance, Clarke et al. demonstrated the prevalence of physician-diagnosed food allergies in Canada was stable at 5.9% in 2010 to 6.1% in 2016, while self-reported food allergies increased from 7.1 to 9.3% over the same timeframe [17]. Similarly, self-reported food allergies among adults in the USA was found to be 19% (of 26 million surveyed) while the proportion of physician-diagnosed food allergies was only 10% [18]. The increasing prevalence of allergic conditions likely influences patient experience and thereby satisfaction with proposed diagnoses and treatment plans for children and their parents alike. Improving awareness of the prevalence of allergic disease and signs/symptoms of true food allergy at the level of the general population as well as healthcare providers is crucial. This will enable advocacy for better support for Primary Care Providers in guiding allergic diagnosis and management, stronger access to specialist care through additional trainees in pediatric allergy, streamlined referral processes, and labelling of allergens for the public. An example is the www.allergycheck.ca resource tool that the UBC Division of Pediatric Allergy & Immunology developed to educate the public on when signs/symptoms are compatible with true food allergy, and when to seek specialist referral [19].

Also contributing to the complexity of these visits is the observation that the average number of allergic conditions per patient is related to the number of opinions sought, suggesting more complex cases may require a more collaborative approach to providing care. However, currently there is a lack of support for primary care physicians and an immense difficulty in accessing the appropriate Allergist for their patients. Allergists in British Columbia have increased their capacity for visits, from 1218 visits per Allergist in 2015–2016, to 1735 in 2019–2020. Additionally, a fellowship training program has been initiated to increase the number of trained Allergists in BC. Despite these changes access to fellowship-trained Allergists in both BC and Canada remains difficult and marred by long waitlists.

Improving care for pediatric allergy patients requires stronger support for general Pediatricians and Primary Care Providers. Recently, a study by Lai et al. discussed the implementation of a novel “eConsult” program wherein Canadian Primary Care Providers electronically connect with pediatric specialists to submit “elective” questions, for which they received guidance within 1 day as opposed to the average of 132 days for a face-to-face referral [20]. Additionally, local and national educational initiatives such as continuing practice conferences provide up-to-date allergy information for physicians and allied health professionals. Another solution could be to build care networks of physicians with additional training who can participate in evidence-based care for the growing number of allergy patients. To accommodate these changes, centralized referral systems like those developed for penicillin allergy triage would allow for improved support and direct access to Allergists with current capacity for referrals [21]. Further, this would enable more capacity for oversight and formal guidance of these networks by certified Allergists. Together, by improving support and access for referring providers, it may be possible to improve patient satisfaction in initial consultations, one of the most common motivational factors for seeking subsequent opinions. Only through this holistic, systems-level approach can we work towards better supporting trained Allergists and Primary Care Providers alike, together aligned in the goal of accessible, evidence-based care for Canadian patients with allergic disease.

## Conclusion

In a retrospective chart-review analysis of multiple-opinion consults completed at BCCH Allergy Clinic over a 1-year time frame, we find that 20.4% of new visits previously sought allergy assessment from another healthcare provider. The predominant rationale for seeking multiple-opinions included a desire to see a fellowship-trained Allergist, as well as dissatisfaction with previous services, and frustration with current wait-times. Despite the understandable frustration, these multiple-opinion consults place a large demand on both healthcare providers and the already-overwhelmed system, ultimately imposing further barriers to patients accessing the care they need.

While our study highlights the prevalence of multiple-opinion consults at the BCCH Allergy Clinic, it can be used to advocate for and drive actionable change at the national level. Future studies will examine specific reasons for patient dissatisfaction with prior consults and explore patient-generated change ideas through patient journey mapping sessions and subsequent assessment of patient satisfaction. With these data, advocacy at the systemic level becomes possible, from bolstering education of and support for patients, families and referring providers, to enacting policy enabling additional trainees in these high-demand fields. Together, we hope to both improve access for our patients, thereby achieving the goal of implementing measures to better prevent allergic disease in children.

## Data Availability

The datasets used and analyzed during the above study are available from the corresponding author on reasonable request.

## Abbreviations

BCCH: British Columbia Children’s Hospital
EMR: Electronic Medical Record
sIgE: Serum-specific IgE levels
